# The Associations of Serum Lipids with Vitamin D Status

**DOI:** 10.1371/journal.pone.0165157

**Published:** 2016-10-21

**Authors:** Ying Wang, Shaoyan Si, Junli Liu, Zongye Wang, Haiying Jia, Kai Feng, Lili Sun, Shu Jun Song

**Affiliations:** 1 Center for Special Medicine and Experimental Research, 306th Hospital of PLA, Beijing, P. R. China; 2 Center for Physical Examination, 306th Hospital of PLA Beijing, P. R. China; Universitat de Lleida-IRBLLEIDA, SPAIN

## Abstract

**Aims:**

Vitamin D deficiency has been associated with some disorders including cardiovascular diseases. Dyslipidemia is a major risk factor for cardiovascular diseases. However, data about the relationships between vitamin D and lipids are inconsistent. The relationship of vitamin D and Atherogenic Index of Plasma (AIP), as an excellent predictor of level of small and dense LDL, has not been reported. The objective of this study was to investigate the effects of vitamin D status on serum lipids in Chinese adults.

**Methods:**

The study was carried out using 1475 participants from the Center for Physical Examination, 306 Hospital of PLA in Beijing, China. Fasting blood samples were collected and serum concentrations of 25(OH)D, total cholesterol (TC), triglyceride (TG), high density lipoprotein cholesterol (HDL-C) and low density lipoprotein cholesterol (LDL-C) were measured. AIP was calculated based on the formula: log [TG/HDL-C]. Multiple linear regression analysis was used to estimate the associations between serum 25(OH)D and lipids. The association between the occurrences of dyslipidemias and vitamin D levels was assessed by multiple logistic regression analysis. Confounding factors, age and BMI, were used for the adjustment.

**Results:**

The median of serum 25(OH)D concentration was 47 (27–92.25) nmol/L in all subjects. The overall percentage of 25(OH)D ≦ 50 nmol/L was 58.5% (males 54.4%, females 63.7%). The serum 25(OH)D levels were inversely associated with TG (β coefficient = -0.24, p < 0.001) and LDL-C (β coefficient = -0.34, p < 0.001) and positively associated with TC (β coefficient = 0.35, p < 0.002) in men. The associations between serum 25(OH)D and LDL-C (β coefficient = -0.25, p = 0.01) and TC (β coefficient = 0.39, p = 0.001) also existed in women. The serum 25(OH)D concentrations were negatively associated with AIP in men (r = -0.111, p < 0.01) but not in women. In addition, vitamin D deficient men had higher AIP values than vitamin D sufficient men. Furthermore, the occurrences of dyslipidemias (reduced HDL-C, elevated TG and elevated AIP) correlated with lower 25(OH)D levels in men, whereas the higher TC and LDL-C associated with higher 25(OH)D levels in women.

**Conclusion:**

It seems that the serum 25(OH)D levels are closely associated with the serum lipids and AIP. Vitamin D deficiency may be associated with the increased risk of dyslipidemias, especially in men. The association between vitamin D status and serum lipids may differ by genders.

## Introduction

Vitamin D is an essential fat-soluble vitamin with multiple functions. The main source of vitamin D in humans is exposure of the skin to sunlight. In skin 7-dehydrocholesterol can be converted to previtamin D_3_ after absorbing solar ultraviolet B radiation and then sequentially hydroxylated into 25(OH)D and 1,25-dihydroxyvitamin D_3_ (an active form) by hydroxylases in the liver and kidney. Vitamin D can be also ingested in the diet or by oral supplements [1]. Besides its classical physiological function of regulation of calcium and bone metabolism, vitamin D is suggested to have many other functions such as modulating immune function, anti-inflammatory activity, suppressing the rennin-angiotensin system and reducing insulin resistance [2–5]. Currently, vitamin D deficiency has been suggested to be associated with some cardiovascular health problems. Low levels of 25(OH)D are independently associated with increased mortality in subjects with cardiovascular disease (CVD) [6]. A meta-analysis of observational studies has shown that decreases in 25(OH)D by 16 ng/dL confer a 16% greater risk for hypertension [7]. A multitude of observations suggest that raising blood levels of 25(OH)D reduces the risk of hypertension, stroke and myocardial infarction [5, 8, 9]. Lipid/lipoprotein abnormalities which refers to raised levels of TC, TG and LDL-C and decreased levels of HDL-C have been identified to be important risk factors of atherosclerosis and CVD [10]. It has been confirmed that lowering of serum cholesterol results in a reduction in cardiovascular morbidity [11]. Whether vitamin D could influence CVD by affecting lipid profile has not been thoroughly studied. Previous studies have suggested that there is a relationship between 25(OH)D levels and serum lipids. However, the results are inconsistent. A cross-sectional study by Jorde et al showed that there were positive associations between serum 25(OH)D levels and TC, HDL-C and LDL-C and a negative association between serum 25(OH)D and TG among 8018 subjects in Norway [12]. Data from the study of Gaddipati VC suggests that serum 25(OH)D levels are negatively associated with TC, TG and LDL-C, and positively associated with HDL-C in Americans [13]. In addition, small and dense LDL (sdLDL) is found to deposit more readily on the arterial wall compared to LDL-C [14]. AIP (log[TG/HDL-C]), as an excellent predictor of levels of sdLDL-C, has been reported to correlate to atherosclerosis and coronary artery disease (CAD) [15]. The potential of AIP to predict cardiovascular risk has been shown in some studies [15, 16]. However, to date, there is no data reported about the relationship between vitamin D and AIP. Therefore the present study was to investigate the relationship of serum 25(OH)D with serum lipids and AIP.

## Subjects and Methods

### Subjects

**Fig 1:**
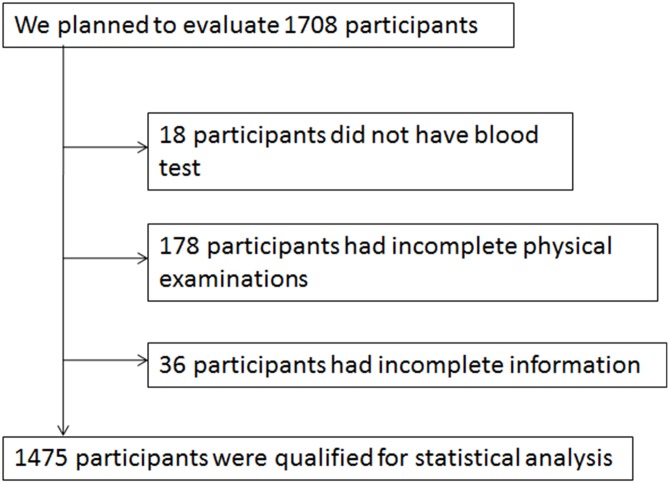
Flow diagram of participant enrollment.

This study was conducted in participants recruited from the Center for Physical Examination, 306 Hospital of PLA in Beijing, China from August 2013 to December 2013, and with no history of malignancies, myocardial infarction, stroke, diabetes, severe liver diseases, kidney disease, and other diseases that affect serum vitamin D levels such as Cushing syndrome, hyperparathyroidism and hyperthyroidism. We planned to evaluate 1708 participants, but only 1475 were analyzed (Fig 1). We also excluded individuals who had supplementation of vitamin D, vitamin D analogues, and any drugs that could affect calcium and phosphorus metabolism and lipid lowering drugs. Authors did not have access to the identifying information of participants during or after data collection.

### Anthropometric and laboratory measurements

The routine measurement of weights (at least to 0.1 kg) and heights (at least to 0.1 cm) was determined by a height and weight measurement instrument. Body mass index (BMI) was calculated based on the formula: BMI = weight (kg)/height (m)^2^.

Fasting blood samples were collected and, centrifuged, and serum was stored at -80°C until use. TC, TG, HDL-C and LDL-C were determined by an enzymatic colorimetric method using an automatic biochemical analyzer [17] (OlympsAu640; Shimotogari, Nagizumi-Chu, Sunto-Gun, Shizuoka, Japan). The intra- and inter-assay coefficients of variation (CVs) for TC were <4% and <5%, respectively. The CVs for TG, LDL-C and HDL-C were <5% (intra-assay) and <6% (inter-assay). The cut-off values of dyslipidemia for TC, TG, LDL-C and HDL-C were set at > 5.7 mmol/L, > 1.7 mmol/L, > 3.36 mmol/L, and < 0.9 mmol/L respectively. These values are normally used as references for our hospital to diagnose dyslipidemia. AIP value was calculated as the logarithm to the base 10 of the ratio of TG to HDL-C (log[TG/HDL-C]). DobiásováoviDL-C]). DobiDobise values are normally used as references for our hospital to diag [18]. Therefore, AIP > 0.15 was regarded as abnormal value in the present study. 25(OH)D, as the metabolizing and the primary circulating form of vitamin D, can be regarded as the best indicator of serum vitamin D status, due to its unique structure, which possesses long half-life and is stable [19]. Serum concentration of 25(OH)D was determined using an enzyme-linked immunosorbent assay kit following the manufacturer's instructions [20] (Immunodiagnostic Systems Ltd, Boldon, Tyne & Wear, United Kingdom). The absorbance was read at 450 nm using a microplate reader (BIO-RAD 550, BioTek, Vermont, USA). The intra-assay CV and inter-assay CV were 4.6% and 6.7% respectively. Although there has been debate over which level of serum 25(OH)D reflects optimum vitamin D status, the Institute of Medicine concluded that the serum 25(OH)D levels > 50 nmol/L could cover the requirements of at least 97.5% of the population which was based on assuring bone health [21]. In our study 25(OH)D levels ≦ 50nmol/L and 25(OH)D levels > 50nmol/L were considered as vitamin D deficiency and vitamin D sufficiency respectively.

### Ethics statement

The protocol of this study was approved by the Ethics Committee of the 306 Hospital of Chinese People's Liberation Army. Written consent was obtained from all participants.

### Statistical analysis

Statistical analysis was performed using Statistical Product and Service Solutions (SPSS) software version 16.0 (SPSS Inc, Chicago, IL, USA). Descriptive characteristics for participants were presented as medians (5th-95th) for non-normally distributed continuous variables and expressed as means (SD) for normally distributed continuous variables which were determined by a Kolmogorov-Smirnov test. The Mann-Whitney U test (non-normally distributed variables), t test (normally distributed variables) and the chi square test (categorical variables) were used to compare the differences between groups. Multiple linear regression analysis was carried out to estimate the associations between serum 25(OH)D concentration (independent variable) and serum lipids (dependent variables: TC, TG, LDL-C, HDL-C). Effect modification by sex was evaluated by stratified analyses of covariance. The test for statistical interaction was added with sex × TC, sex × TG, sex × LDL-C and sex × HDL-C into the multiple regression. TG and HDL-C were transformed from non-normally distributed variables to normally distributed variables after a logarithmic transformation. We analyzed the association between 25(OH)D and AIP using Spearman's rank correlation coefficient. Logistic regression analysis was further used to evaluate the associations between the occurrences of dyslipidemias and 25(OH)D levels and data was expressed as odds ratios (OR) with 95% confidence intervals (CI). All *p* values were two tailed and < 0.05 were considered to be statistically significant.

## Result

### General characteristics

Clinical and anthropometric characteristics of participants were summarized in Table 1. The overall percentage of vitamin D deficiency was 58.5% (males 54.4%, females 63.7%). While, males had significantly higher values in age, BMI, TG, LDL-C, 25(OH)D and lower HDL-C compared to females (Table 1).

**Table 1:** Subject characteristics.

Variables	All (n = 1475)	Men (n = 829)	Women (n = 646)	*P* -value
Age	39 (24–64)	40 (25–64.5)	38 (24–64)	<0.001^b^
BMI (kg/m^2^)	24.57 (3.67)	25.69 (20.24–31.51)	22.38 (18.36–28.87)	<0.001^b^
TC (mmol/L)	4.73 (3.49–6.31)	4.78 (0.85)	4.76 (0.86)	0.58^a^
TG (mmol/L)	1.17 (0.51–3.61)	1.39 (0.59–4.35)	0.93 (0.47–2.49)	<0.001^b^
LDL-C (mmol/L)	3.01 (0.79)	3.12 (0.80)	2.86 (0.76)	<0.001^a^
HDL-C (mmol/L)	1.29 (0.89–2.01)	1.18 (0.85–1.74)	1.47 (1.01–2.18)	<0.001^b^
25(OH)D (nmol/L)	47 (27–92.25)	48 (28–97)	43 (25–85)	<0.001^b^
AIP	-0.02 (0.32)	0.10 (0.29)	-0.18 (0.28)	<0.001^a^

BMI, body mass index; TC, Total cholesterol; TG, triglycerides; LDL-C, low-density lipoprotein; HDL-C, high-density lipoprotein; 25(OH)D, 25-hydroxyvitamin D; AIP, atherogenic index of plasma.

^a^Student’s t–test was used to compare the mean values of normally distributed variables.

^b^The Mann-Whitney U test was used to examine non-normally distributed variables.

P values were used to assess the differences between males and females.

Age, TG, HDL-C and 25(OH)D in men, women and all participants, BMI in men and women and TC in all participants were presented as medians (5th-95th). BMI in all participants, TC in men and women as well as LDL-C and AIP in men, women and all participants were expressed as means (SD).

### The association between serum 25(OH)D concentrations and lipids in men and women

Multiple regression analysis was used to assess the associations between serum 25(OH)D concentrations and lipids (Table 2). In males 25(OH)D concentrations were negatively associated with TG and LDL-C and positively associated with TC after adjusting for age and BMI. Per 10 nmol/L increase in serum 25(OH)D was associated with decreases of 1.74 mmol/L in TG and 0.34mmol/L in LDL-C and an increase of 0.35 mmol/L in TC. We also found an apparently negative association between 25(OH)D and LDL-C and a positive association between 25(OH)D and TC in women after adjustment for age and BMI. Each 10 nmol/L increment in 25(OH)D concentration was associated with a decrease of 0.25 mmol/L in LDL-C and an increase of 0.39 mmol/L in TC in women. Whereas there was no significant association between 25(OH)D and HDL-C either in males or in females. Substantial sex differences existed, the effect modification by sex was not significant with respect to TC, TG, LDL-C and HDL-C (p > 0.05).

**Table 2:** The association between serum 25(OH)D concentrations and serum lipids in males and females after adjusting for age and BMI.

	Men (n = 829)		Women (n = 646)	
Variables	β ariablesati	p-value	β coefficient	p-value
TC	0.35	0.002	0.39	0.001
TG	-0.24	<0.001	-0.09	0.118
LDL-C	-0.34	<0.001	-0.25	0.01
HDL-C	-0.07	0.196	-0.09	0.176

TC, Total cholesterol; TG, triglycerides; LDL-C, low-density lipoprotein; HDL-C, high-density lipoprotein.

25(OH)D, TG and HDL-C were log transformed for the analysis.

βcoefficient is a standardized coefficient in multiple linear regression analysis.

### The correlation between vitamin D status and AIP

**Fig 2:**
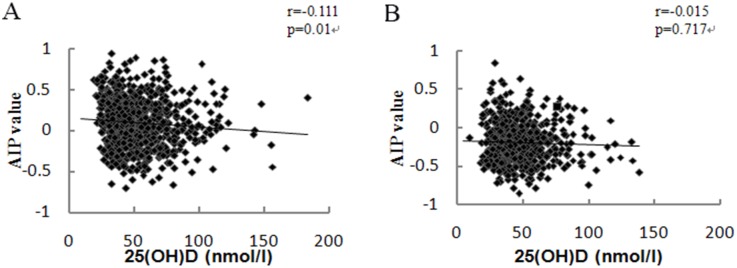
The correlation between 25(OH)D and AIP. Scatter plot showing the relations between serum 25(OH)D concentrations and AIP in men (A) and women (B).

The association between vitamin D and AIP was analyzed using Spearman's Rank Correlation Coefficient. The serum 25(OH)D concentrations were negatively associated with AIP in men. However there were no significant associations between 25(OH)D concentrations and AIP in women (Fig 2).

### The comparison of lipid profiles at different vitamin D status

To investigate whether the relationship between serum 25(OH)D and serum lipids differs by vitamin D status, we categorized the participants into two subgroups of vitamin D deficiency and vitamin D sufficiency. The results showed that TG and AIP were significantly higher in men with vitamin D deficiency compared to those in men with vitamin D sufficiency (Table 3). Furthermore, The percentages of dyslipidemias and abnormal AIP values by vitamin D status were also presented in Table 3. The incidences of elevated TG, reduced HDL and elevated AIP were higher in vitamin D deficient men than those in vitamin D sufficient men. However, there were no significant differences with these parameters between the groups in females.

**Table 3:** The comparison of characteristics at different vitamin D status in males and females.

	Men			Women		
	25(OH)Dent vitam (451)	25(OH)D>50nmol/L (378)	p-value	25(OH)DLnt vitam (413)	25(OH)D>50nmol/L (233)	p-value
Age	39 (24–62)	40 (25–67)	0.01^b^	37 (24–64.35)	39 (24–63)	0.59 ^b^
BMI (kg/m2)	25.93 (3.37)	25.73 (3.30)	0.40^a^	22.2 (18.27–29.06)	22.72 (18.41–28.32)	0.23 ^b^
TC (mmol/L)	4.80 (0.85)	4.76 (0.85)	0.59 ^a^	4.71 (0.88)	4.83 (0.83)	0.87 ^a^
TG (mmol/L)	1.51 (0.596–4.4)	1.31 (0.59–4.25)	0.01 ^b^	0.93 (0.48–2.16)	0.93 (0.46–2.64)	0.36 ^b^
LD-L-C (mmol/L)	3.15 (0.76)	3.08 (0.85)	0.18 ^a^	2.79 (1.65–4.18)	2.84 (1.72–4.23)	0.38 ^b^
HDL-C (mmol/L)	1.17 (0.81–1.73)	1.19 (0.88–1.75)	0.13 ^b^	1.46 (0.99–2.21)	1.48 (1.06–2.11)	0.48 ^b^
AIP	0.12 (0.30)	0.07 (0.28)	0.01 ^a^	-0.18 (0.27)	-0.17 (0.29)	0.59 ^a^
Elevated TC %(n)	13.08% (59)	16.74% (39)	0.08	12.34% (51)	16.73% (39)	0.13^c^
Elevated TG %(n)	43.46% (196)	33.86% (128)	0.01	11.86% (49)	15.88% (37)	0.15^c^
Elevated LDL-C %(n)	9.97% (45)	5.82% (22)	0.24	22.76% (94)	26.61% (62)	0.29^c^
Reduced HDL-C %(n)	36.14% (163)	32.01% (121)	0.03	2.18% (9)	0.89% (2)	0.34^c^
Elevated AIP %(n)	46.12% (208)	37.83% (143)	0.02	13.38 (47)	11.59% (27)	0.52^c^

25(OH)D, 25-hydroxyvitamin D; BMI, body mass index; TC, Total cholesterol; TG, triglycerides; LDL-C, low-density lipoprotein; HDL-C, high-density lipoprotein; AIP, atherogenic index of plasma.

Differences in data between the different vitamin D groups was analyzed by ^a^student’s t–test for normally distributed continuous variables, ^b^Mann-Whitney U test for non-normally distributed continuous variables or ^c^chi square test for the categorical variables.

Age, TG and HDL-C in men, women and all participants, and BMI and LDL-C in women were presented as medians (5th-95th). BMI and LDL-C in men and TC and AIP in men, women and all participants were expressed as means (SD).

### The association between the occurrences of dyslipidemias and 25(OH)D levels

Unadjusted OR and age and BMI adjusted OR for dyslipidemias and elevated AIP by serum 25(OH)D levels in men and women were presented in Table 4. After adjusting for age and BMI, the OR for the elevated TG, reduced HDL-C and elevated AIP decreased significantly in vitamin D sufficient men compared with vitamin D deficient men. In contrast, in women, the OR for the incidence of dyslipidemias or elevated AIP showed no significant difference between vitamin D sufficient group and vitamin D deficient group.

**Table 4:** Odds ratio of dyslipidemias and elevated AIP by serum 25(OH)D levels in men and women.

	Men			Women		
	25(OH)D in men and wom	25(OH)D>50nmol/L (378)	p-value	25(OH)DL (378) and wom	25(OH)D>50nmol/L (233)	p-value
Elevated TC						
Unadjusted	1.000	0.943 (0.626–1.422)	0.781	1.000	1.427 (0.908–2.242)	0.123
Adjusted	1.000	0.909 (0.600–1.376)	0.652	1.000	1.423 (0.901–2.278)	0.129
Elevated TG						
Unadjusted	1.000	0.666 (0.502–0.884)	0.005	1.000	1.402 (0.885–2.223)	0.150
Adjusted	1.000	0.612 (0.450–0.831)	0.002	1.000	1.453 (0.887–2.382)	0.138
Elevated LD-L-C						
Unadjusted	1.000	0.832 (0.623–1.111)	0.212	1.000	1.230 (0.849–1.782)	0.273
Adjusted	1.000	0.838 (0.625–1.123)	0.237	1.000	1.223 (0.825–1.814)	0.315
Reduced HDL-C						
Unadjusted	1.000	0.558 (0.328–0.947)	0.031	1.000	0.389 (0.083–1.814)	0.229
Adjusted	1.000	0.570 (0.333–0.975)	0.04	1.000	0.426(0.089–2.035)	0.285
Elevated AIP						
Unadjusted	1.000	0.711 (0.538–0.939)	0.016	1.000	1.021 (0.617–1.688)	0.937
Adjusted	1.000	0.667 (0.492–0.905)	0.009	1.000	1.023 (0.600–1.746)	0.932

25(OH)D, 25-hydroxyvitamin D; TC, Total cholesterol; TG, triglycerides; LDL-C, low-density lipoprotein; HDL-C, high-density lipoprotein; AIP, atherogenic index of plasma.

The OR and p-value for the prevalence of dyslipidemias and elevated AIP between the two levels of serum 25(OH)D were analyzed using logistic regression after adjustment for age and BMI. Data are presented as OR (95% CI).

## Discussion

Vitamin D is a multifunction vitamin. Vitamin D deficiency has been suggested to be associated with a number of conditions including cardiovascular disease [22, 23]. The dyslipidemia which is characterized by elevations of LDL, raised TG, and decreases in HDL-C has been well-established as a CVD risk factor [24, 25]. Vitamin D may impact cardiovascular health through influencing serum lipids. Our results showed that in male participants, serum 25(OH)D concentrations had a strong negative association with TG and LDL-C after adjusting relevant confounders such as age and BMI. These findings add to the evidence that serum lipids correlate with levels of 25(OH)D, a finding that has been reported in previous studies [26–28].

We also found that vitamin D deficient men had higher TG values compared to vitamin D sufficient men. Moreover, the higher incidences of elevated TG and reduced HDL-C were associated with the lower 25(OH)D levels in males. These results further suggest that vitamin D deficiency may be associated with the increased risk of dyslipidemias. The associations between vitamin D and serum lipids have been extensively studied in different populations. Jungert A et al found that 25(OH)D levels were positively associated with HDL-C and inversely associated with TC, HDL-C, LDL-C:HDL-C and TC:HDL-C among the elderly women in Germany [28]. Data from the study of Karhapää et al suggests that serum 25(OH)D levels are negatively associated with TC, TG and LDL-C in middle-aged Finnish males [27]. The findings from Sun’s study of 136 Japanese males aged 20–79 years indicate that vitamin D is inversely correlated with TG and LDL-C:HDL-C [26]. Results from the study of Skaaby et al showed that a 10 nmol/l higher level of 25(OH)D was associated with a decrease in TG (0.52%) and VLDL-C (0.66%) in Danish adults [29]. They also found that there was a causal effect of higher vitamin D status on a more favorable lipid profile [30]. These results suggest that associations between the levels of vitamin D and serum lipid profiles exist among different populations and maintaining vitamin D sufficiency seems to have a beneficial effect on serum lipids. This potential association still needs further study and clarification.

sdLDL-C is more easily to deposit on the arterial wall by binding to glycoprotein compared to LDL-C [31]. Elevated sdLDL-C is predominantly responsible for cholesterol depositing and decreasing in clearance of LDL-C [14]. What’s more, sdLDL-C prefers to transform into oxidized low density lipoprotein which would be taken up by macrophages to form foam cells. Therefore, sdLDL-C becomes one of the major causative factors of arteriosclerosis and cardiovascular disease [32]. AIP, as a transformation of TG/HDL-C, was first introduced by DobiásováoviDobieda transformation of TG/HDL-C, was first <Year>sdLDL-C [33]. In addition, elevating TG and/or decreasing HDL-C could cause AIP to rise. Hypertriglyceridemia and/or hypo-HDL cholesterolemia as special types of dyslipidemia are thought to be high risk factors for atherosclerosis and CAD [34, 35]. AIP has been shown to be a more useful marker of atherogenicity and cardiovascular risk than single LDL-C or TC [36, 37]. AIP, as an index of dyslipidaemia to predict the risk of developing atherosclerosis and CVD, has been applied in some studies [15, 16]. In our study we observed that levels of 25(OH)D had a negative association with AIP and elevated AIP values were associated with lower 25(OH)D levels in males. The data further suggests that the improvement of vitamin D status may have favorable potential in reducing the risk of dyslipidemias.

However, how vitamin D influences lipid profile is not clear yet. Previous data has suggested that increasing intestinal calcium absorption could reduce synthesis and secretion of hepatic TG [38]. Vitamin D could inhibit synthesis and secretion of TG through stimulating intestinal calcium absorption. It has also been suggested that increased level of intestinal calcium could reduce intestinal absorption of fatty acid due to the formation of insoluble calcium-fatty complexes. Serum levels of LDL-C would be reduced by the decreased absorption of fat, particularly saturated fatty acids [39]. In addition, calcium could promote the conversion of cholesterol into bile acids and thereby reduce the level of cholesterol [40]. Other studies have proved that high level of parathyroid hormone (PTH) could result in TG elevating and higher concentrations of 25(OH)D suppress serum PTH levels [41, 42]. Therefore, vitamin D could influence TG concentrations by regulating PTH levels. In addition, previous studies have provided a strong evidence that vitamin D deficiency may be associated with impaired b-cell function and insulin resistance which could affect lipoprotein metabolism and lead to an increase in TG level and a decrease in HDL-C level [43–45]. In addition, vitamin D has been suggested to be involved in lipid metabolism such as the synthesis of bile acid in the liver [46], suggesting that vitamin D may affect the regulation of lipids directly.

In the present study, we observed that the associations between serum 25(OH)D levels and serum lipids were more pronounced in males than in females, which was also found in Yin’s study [47]. Previous studies have shown that serum 25(OH)D levels have no significant relationship with lipid profile among postmenopausal women [48]. The difference of hormone and hormonal sensitivity of the target tissue between genders could impact lipid metabolism differently. Life style difference such as smoking, alcohol consumption, sun exposure and physical activity may also contribute to the dissimilar results between males and females.

In addition, the results in Table 2 showed that 25(OH)D was positively associated with TC and negatively associated with LDL-C, which was also showed in Bolland’s study [49]. Total cholesterol consists of several components including LDL-C, HDL-C and VLDL-C [50]. The interactions between 25(OH)D and each of these components might be different. The association between TC and 25(OH)D is a composite outcome of the associations between 25(OH)D and each of these components in TC. Therefore, the association between 25(OH)D and TC could be different from the association between 25(OH)D and any one of the cholesterol components.

In the current study the percentage of serum 25(OH)D ≦ 50 nmol/L in participants was 58.5%. A previous study has shown that the prevalence of vitamin D deficiency accounted for 66.2% among middle-aged Chinese population [47]. A study from the United States of America demonstrated that half of the subjects had vitamin D deficiency [49]. More recently, A Vitezova et al showed 57% of the participants in a Netherlands study had vitamin D deficiency [51]. A study from Sun X et al showed that 78.7% of Japanese male participants were 25(OH)D-deficiency [26].These results suggest that vitamin D deficiency is a common problem in many populations.

## Limits and Strength

Several limitations of this study should be considered when interpret these results. First of all, as with the nature of all the cross-sectional studies, the results could not be reflection of causality. Further investigations are needed to establish the causal relationship between vitamin D and serum lipid levels. For example, interventional studies should be taken into consideration. Secondly, all participants in our study were from Beijing, which limits the generalized application of the results to the whole country. Thirdly, although our study took into account the potential confounding factors (age and BMI), there were still some other confounding variables that may have an influence on the relationship between vitamin D and serum lipids such as waist circumference, sun exposure, physical activity, smoking, etc. In addition, the associations between vitamin D and lipids were possibly influenced by levels of calcium and parathyroid hormone which we did not measure. Despite the above limitations, to the best of our knowledge, the present study is the first data evidence for investigating the association of serum 25(OH)D levels with AIP.

## Conclusions

In summary, Vitamin D deficiency is common among adults living in Beijing. Serum 25(OH)D levels seem correlated with the serum lipids. Vitamin D deficiency may be associated with the increased risk of dyslipidemias. The relationship of vitamin D status and serum lipids may differ by genders. Large randomized, placebo-controlled trials will be required to better-understand the relationship between vitamin D and serum lipid profiles.

## Supporting Information

S1 Appendix The dataset used in this study. (XLSX)
